# FedMal-XAI: an explainable federated vision transformer leveraging knowledge distillation for privacy-preserving malaria detection

**DOI:** 10.3389/fpubh.2026.1769078

**Published:** 2026-03-26

**Authors:** Tofael Ahmed Bhuiyan, Abdur Rahman, Fokrul Islam Khan, Farzan Majeed Noori, Abdul Kadar Muhammad Masum

**Affiliations:** 1 Computational Intelligence Lab, Southeast University, Dhaka, Bangladesh; 2 College of Business, Westcliff University, Irvine, CA, United States; 3 Department of Informatics, University of Oslo, Oslo, Norway; 4 Department of Computer Science and Engineering, Southeast University, Dhaka, Bangladesh

**Keywords:** explainable AI, federated learning, malaria detection, medical imaging, privacy preservation, vision transformers

## Abstract

Plasmodium parasites are the cause of malaria, a deadly illness that continues to pose a serious danger to world health, especially in areas with low resources where subjectivity, complexity, along with privacy issues make it difficult to employ traditional diagnostic techniques like microscopy and quick diagnostic testing. To overcome these specific challenges of diagnostic subjectivity, logistical complexity, and data privacy, this paper suggests a privacy-preserving federated learning system that uses sophisticated Vision Transformers (ViTs) for automated malaria identification from blood smear images. This paper suggests a privacy-preserving federated learning system that uses sophisticated Vision Transformers (ViTs) for automated malaria identification from segmented red blood cell (RBC) images in order to get around these issues. This architecture successfully addresses important privacy and logistical restrictions by enabling cooperative training among decentralized institutions without exchanging sensitive data. Prominent centralized convolutional neural networks (CNNs) are matched in diagnostic accuracy by the federated ViT models, which include ViT-B/16, DeiT-Tiny, Swin-T, and DINOv2. Interestingly, the federated transformer variations outperform the CNN ensemble (ResNet50 + VGG16) with an accuracy of 98.15%, FedDistill-DeiT achieving 97.79%, FedAvg-Swin-T reaching 97.75%, and FedDistill-Swin-T achieving a high ROC-AUC of 0.9977. These findings show that, even in the presence of diverse data distributions, federated Vision Transformers provide a reliable, scalable, and interpretable malaria screening solution that combines high accuracy with solid privacy guarantees.

## 1 Introduction

Malaria continues to be a serious worldwide health burden, impacting millions of people each year. The cause of this fatal disease is the plasmodium parasites and is transmitted through the bite of the female Anopheles mosquitoes infected with the disease. World Health Organization (WHO) estimates that in 2024, malaria in 80 endemic countries accounted for 282 million cases, and approximately 610,000 deaths are caused by this disease, the majority of which occurs in children under five in sub-Saharan Africa (1). Timely and correct diagnosis is important in effective treatment and control of illness because any error or delay in diagnosis stands a high chance of severe effects and death. Rapid diagnostic tests (RDTs) and microscopic examination of blood smears are the major pillars of conventional diagnostic methods. Although microscopy continues to be the gold standard, frequent application in low-resource settings is challenging since it is labor intensive and requires highly skilled labor. Moreover, subjective interpretation of morphological characteristics usually presents inter-observer variability, which threatens the consistency of diagnosis (2, 3). RDTs by contrast frequently find application in the point-of-care; however, they cannot measure the parasite, an essential parameter in clinical decision-making and epidemiological surveillance, and sensitivity and specificity vary between Plasmodium species (4). Furthermore, the diagnostic reliability of RDTs is increasingly threatened by critical biological challenges, such as *pfhrp2* and *pfhrp3* gene deletions, which cause false-negative results even in highly symptomatic patients. Additionally, RDTs are susceptible to the prozone phenomenon, where exceptionally high parasite densities paradoxically yield negative test results, further limiting their dependability in severe cases. These compounding shortfalls in conventional methods emphasize the urgent need for robust, scalable, and automated diagnostic alternatives like advanced deep learning frameworks that remain unaffected by these specific biological anomalies.

Recent paradigms in deep learning and artificial intelligence (AI) have revolutionary opportunities to eliminate the natural constraints of the classical malaria diagnosis. Deep learning, a particular form of machine learning, can automatically learn to make hierarchical representations of features in raw data without the input of any kind of preprocessing. Convolutional neural networks (CNNs) have demonstrated impressive levels of performance in the medical image processing domain, enhancing diagnostic accuracy and efficiency, as well as diagnostic repeatability (5–9). CNNs are able to detect clinically salient features with astonishing accuracy as shown by one of the studies that trained DenseNet-121 architecture with a soft-attention mechanism to view regions of interest. The sensitivity of the study was 99.2 and the specificity was 99.4 (10). Besides the diagnosis of malaria, CNNs have also been utilized in the diagnosis of neurological disease such as Parkinson. Transfer learning, utilizing pre-trained models like VGG16, further enhances performance while reducing computational overhead (11). Collectively, these advancements demonstrate the potential of deep learning to overcome diagnostic bottlenecks in low-resource settings where traditional microscopy often faces challenges with consistency and scalability. Transfer learning, whereby a pre-trained model like VGG16 is used to solve a particular diagnostic task, is another method, which has become a potent approach in biomedical imaging at once. This improves performance, and it lowers the cost of computing (11). Taken together, these developments highlight the immense potential of deep learning to address diagnostic bottlenecks, particularly in low-resource settings where traditional methods often fail to deliver effective results.

In this study, transformer-based architectures are used to automatically identify Plasmodium parasites in segmented red blood cell (RBC) images, introducing a federated learning framework for malaria diagnosis that prioritizes data privacy and interpretability. In order to provide a thorough comparison across configurations, the study includes centralized models (ViT-B/16, DeiT-Tiny, Swin-T, as well as CNN baselines VGG16, ResNet50, EfficientNetB3, and their ResNet50 + VGG16 ensemble) and federated variations (FedAvg and FedDistill applied to transformers and DinoV2). Interestingly, individual CNNs get 97.61% accuracy, while the ResNet50 + VGG16 ensemble attains the greatest centralized performance with 98.15% accuracy. FedAvg-DinoV2 records an accuracy of 97.22% and FedDistill-DinoV2 records 95.81% in federated circumstances. Fed Distill-DeiT and FedAvg-Swin-T are the transformer-based models with the greatest overall accuracy, and the best ROC-AUC.

The NIH Malaria Dataset, which includes 27,558 segmented cell pictures split equally between parasitized and uninfected classes, is used for the experiments. The images have been pre-processed by initially resizing them to 256 × 256 pixels, followed by cropping to 224 × 224 pixels, normalization, and targeted augmentation. Accuracy, precision, recall, F1-score, ROC-AUC, MCC, Cohen’s Kappa, parameter efficiency, and communication cost in federated training are all evaluated. Gradient-based saliency maps and LIME, which consistently emphasize physiologically significant markers like trophozoites, schizonts, and hemozoin deposits, guarantee interpretability.

The findings demonstrate that distillation-based federated methods generalize more effectively than averaging approaches in non-IID environments, reducing false positives and negatives while maintaining communication efficiency. This establishes federated transformers as privacy-preserving, interpretable, and scalable diagnostic tools for malaria, particularly in resource-constrained endemic regions. Future directions include clinical deployment across diverse healthcare infrastructures, multimodal data integration, and optimization for heterogeneous non-IID conditions.

Beyond technological developments, our work contributes to global health goals by providing visible and comprehensible AI tools via saliency maps and LIME, which highlight physiologically important malaria indicators like hemozoin deposits and trophozoites. By developing scalable AI-driven diagnostic tools, the research advances Sustainable Development Goal, which places an emphasis on ensuring healthy lives and fostering well-being for all.

Key contributions of this study:

- Proposed Federated Framework: Transformer versions FedAvg and FedDistill were benchmarked against DinoV2 and CNN baselines.
- Feature Extraction: For effective distillation, use DeiT-Tiny; for hierarchical shifted-window modeling, use Swin-T; and for patch-based global attention, use ViT-B/16.
- Scalability: Enhanced communication cycles and parameter effectiveness for implementation in contexts with limited resources.
- Generalizability: Knowledge distillation and explainable AI are combined to increase applicability for medical imaging jobs that are not IID.

Methodologically, this work goes beyond incremental application by engineering a specialized distillation pipeline that leverages transformer-specific class and distillation tokens to mitigate feature divergence in non-IID medical data. By formalizing a hybrid loss mechanism that balances cross-entropy with temperature-scaled Kullback–Leibler divergence specifically for attention-based backbones, the framework provides a novel pathway for preserving global semantic knowledge while allowing local nodes to adapt to site-specific staining and resolution variations. This paper’s remaining sections are organized as follows: In Section 2, relevant research on deep learning as well as malaria detection is reviewed. The dataset, federated framework, transformer designs, and training setups are described in depth in Section 3. Results and discussion for federated and centralized models, including explainable AI, are presented in Section 4. The primary contributions are outlined in Section 5, while limits and potential avenues for further study are described in Section 6.

## 2 Literature review

This part is a critical assessment of the current status of malaria diagnostics, and the constant limitations of the older methods are demonstrated, with the increasing role of deep learning in overcoming these constraints. Traditional diagnostic strategies, though the most popular, are usually marred by problems of low accuracy, scale and applicability especially in low resources areas. The recent introduction of deep learning and medical imaging has brought about a paradigm shift in the form of data-driven and intelligent diagnostic systems which is opening up numerous opportunities to more effective and accurate malaria detection.

Initial efforts to perform automated malaria diagnosis based on conventional machine learning methods produced only modest results, and this is mainly because of the use of handcrafted feature engineering. A hybrid model comprised of VGG architecture and support vector machine was found to have a classification accuracy of 93.1% (12), whereas its low generalization factors further indicated that more powerful models are necessary. Continuing with the basics, Pan et al. (13) used a LeNet-5-based convolutional neural network to be able to diagnose malaria automatically, highlighting the impact of the heterogeneity of the datasets and their size on a high level of performance. But LeNet-5 was limited in its ability to extract features due to its shallow architecture. A comparison of a variety of CNN architectures such as AlexNet, VGG16, Xception, ResNet50, and DenseNet121 conducted by Rajaraman et al. (14) shows that focused-trained models could be successfully used to extract discriminative features. Regardless of these advantages, inconsistent preprocessing and augmentation procedures limited the strength. Equally, Çinar and Yildirim (15) evaluated eight CNNs and discovered GoogleNet to obtain 96.6% accuracy using Gaussian filtering, but the imbalance of data and poor augmentation hampered generalization.

More research was done to perfect deep learning. Song et al. (16) obtained over 95% accuracy based on transfer learning models, whereas Reddy and Juliet (2019) obtained 95.91% based on ResNet50 but they have not performed more advanced regularization strategies. Soylu (17) noted that MobileNet-V2 reached the accuracy of 96.53%, however, due to the lack of the state-of-the-art models like EfficientNet, it did not improve further. Conventional microscopy as the gold standard was still limited by the need to work, the reliance on experts, and the unequal decisions made by experts (18). The rapid diagnostic tests provide quicker results, but they have variable sensitivity among species of Plasmodium and are not able to separate the density of the parasite (19, 20).

Recent developments are automated microscopy, polymerase chain reactions, and loop-mediated isothermal amplification that have higher sensitivity but depend on infrastructure and expertise resources (21–25). Deep learning, especially transformer-based models, has revolutionized medical imaging, as the models are able to model hierarchical and global image dependencies (26). Self-supervised learning also improves the diagnostic performance in the absence of massive labeled datasets (27). Transformers have now outperformed the traditional ones in terms of malaria detection, with the highest accuracy rating of 97.79%, and guarantee the privacy and scalability of data through federated learning (28–30).

## 3 Materials and methods

**Figure 1:**
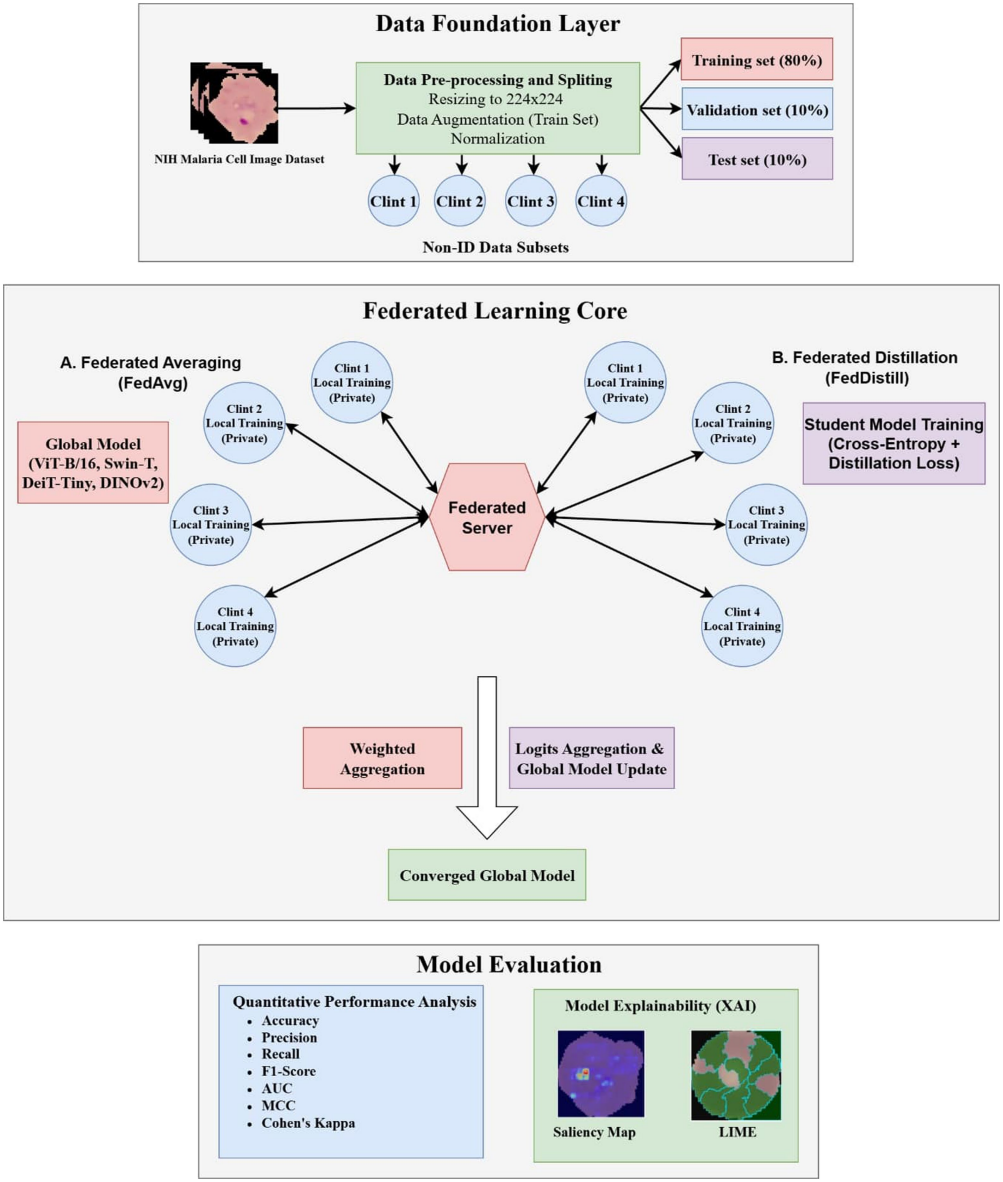
Conceptual diagram of the proposed deep and federated learning system.

Figure 1 presents a malaria detection pipeline that integrates interpretability, assessment, training, and data preparation. Cell images from the publicly accessible NIH Malaria dataset were initially resized to 256 × 256 pixels, acting as a base resolution before subsequent 224 × 224 cropping, and normalized using widely adopted mean and standard deviation values (31). The dataset is available on the United States National Library of Medicine website. Extensive augmentation enhanced model robustness through random cropping to 224 × 224, horizontal and vertical flips, rotations up to 90 degrees, affine changes, color jitter, Gaussian blur, perspective shifts, random erasing, and adaptive resizing. A fixed seed ensured a consistent split into training, validation, and testing groups with a ratio of 70, 15, and 15%. This 70/15/15 distribution was strategically chosen to accommodate the requirements of data-intensive models while maintaining evaluation rigor. Allocating 70% (19,290 images) provides the extensive data volume required to adequately train complex Vision Transformer architectures. Concurrently, the 15% validation (4,134 images) and 15% test (4,134 images) sets offer sufficiently large, independent samples to ensure robust hyperparameter tuning, reliable early stopping, and statistically significant evaluations of the models’ generalization capabilities on unseen data.

Transformer models including ViT B/16, DeiT-Tiny, Swin-T, and DINOv2 were optimized using custom classifier heads, dropout layers, and normalization strategies. Each architecture employed distinct feature extraction depths. Training used AdamW with gradient clipping, label smoothing, cosine annealing, and warm restarts. Federated learning involved 10 global rounds, with each client running 5 local epochs. FedAvg applied weighted aggregation, while FedDistill used knowledge distillation. Centralized CNN benchmarks included VGG16, ResNet50, and EfficientNetB3. Interpretability relied on saliency maps and LIME, which highlighted key parasitic structures. Results were highly reliable.

### 3.1 Experimental data

The experimental study used the NIH Malaria Dataset, which is freely available and comprises 27,558 segmented cell images from thin blood smears collected during the Malaria Screener project (31). Regarding clinical characteristics and patient enrollment, the NIH dataset was sourced from Giemsa-stained thin blood smear slides collected from 150 *Plasmodium falciparum*-infected patients and 50 healthy patients at Chittagong Medical College Hospital, Bangladesh. The patient enrollment and specimen collection were conducted to capture real-world clinical variations, with the disease-related profile strictly focused on *P. falciparum*, which is the most deadly and prevalent malaria-causing parasite in that specific geographic region. Furthermore, the supplementary NM-AIST dataset encompasses blood smears acquired from patients in local Tanzanian health facilities. This dataset introduces distinct clinical staining protocols, varying parasitemia levels, and localized *Plasmodium* species variations endemic to East Africa, thereby providing a robust clinical cross-section for evaluating the federated learning models under real-world, multi-institutional conditions. During the creation of this dataset, following the initial automated cell segmentation, expert slide readers meticulously performed manual curation. Each individual red blood cell (RBC) image was visually examined and annotated by these experts to confidently classify them into parasitized or uninfected categories, thereby establishing a highly reliable and clinically validated ground truth for our model training. The dataset is balanced with 13,779 images each for parasitized and uninfected classes, enabling unbiased model training without extra augmentation for class balance. To further validate the robustness of the federated learning framework in a true multi-institutional setting, we incorporated The Nelson Mandela African Institution of Science and Technology (NM-AIST) Malaria Dataset. This additional dataset introduces real-world heterogeneity, simulating a second distinct client with different staining and acquisition characteristics compared to the NIH dataset (32). All originally varied-resolution images were resized to 256 × 256 pixels to align with transformer requirements. The data were split into training with 19,290 samples, validation with 4,134, and testing with 4,134 using a fixed seed of 42 for reproducibility. A binary label scheme was applied, where parasitized cells were assigned 1 and uninfected cells 0. Centralized models including VGG16, ResNet50, EfficientNetB3, their ensemble, and transformer-based architectures were trained and evaluated using these partitions. To strictly validate the system against cross-institutional domain shift and device heterogeneity, we incorporated the NM-AIST Malaria Dataset as a distinct client source. Unlike the NIH dataset, the NM-AIST samples were acquired using different microscope sensors and staining protocols. This setup moves beyond simple data partitioning to a realistic Federated Learning scenario where clients possess non-IID data with inherent acquisition differences.

For simulation purposes, the training portion was divided into four non-IID clients, considering heterogeneous data distributions across healthcare sites for federated learning. To formally simulate non-IID conditions (Label Skew), we partitioned the data using a Dirichlet distribution *Dir(α)*. For each client *k*, the proportion of samples of class *c*, denoted as *p*_*c,k*,_ is sampled such that p*_c,k_* ∼ *Dir(α)*, where *α* = 0.5 controls the degree of heterogeneity. Furthermore, the inclusion of the distinct NM-AIST dataset introduces Feature Skew, representing a natural covariate shift. The validation and test sets remained centralized to ensure a fair assessment. In this federated distillation setup, each client updates its model using soft labels extracted from the previous global model. The distillation process uses a temperature of two, with a hybrid loss of cross-entropy and distillation loss weighted at 0.4, which enhanced generalization and communication efficiency with improved privacy.

### 3.2 Data pre-processing

A unified preprocessing pipeline was followed to maintain consistency between both federated and centralized models. All images were converted to RGB, initially resized to 256 × 256 pixels, and subsequently cropped to a final target resolution of 224 × 224 pixels to match the required network input dimensions. Standardized real-time augmentations- including 90-degree rotations, horizontal/vertical flips, and color jitter-were applied to the training set to bolster model robustness. To maintain consistency across experiments, images were converted to tensors and normalized using ImageNet-derived mean and standard deviation values, ensuring a uniform input distribution for both centralized and federated architectures. Aggressive real-time augmentations, such as random resized crop, 90-degree rotation, horizontal and vertical flips, color jitter, affine transformations with shear and scale, and Gaussian blur, were applied during training. These transformations were applied exclusively to the 19,290 training images on-the-fly, leaving the validation, test, and external NM-AIST datasets strictly unaltered for fair evaluation. The fundamental justification for this aggressive augmentation strategy stems from the data-hungry nature of Vision Transformers, which lack the inductive biases of CNNs and are highly susceptible to overfitting on moderately sized medical datasets. Furthermore, augmentations such as color jitter and Gaussian blur explicitly simulate real-world variations in blood smear microscopy-including inconsistent staining intensities, varying illumination, and slight focus shifts-thereby ensuring the models learn generalized, invariant features that are crucial for robust federated learning across diverse clinical sites. Images were further converted to tensors and normalized after augmentation using the ImageNet mean and standard deviation values. For the validation and test sets, images were simply resized to 256 × 256 pixels and center-cropped to 224 × 224 pixels, followed by normalization, ensuring that no random spatial augmentations were applied during evaluation.

To set up federated learning, the training data were split across four clients, each receiving approximately 4,823 samples, while validation and testing remained centralized. This allowed fair comparisons among ViT B/16, DeiT Tiny, Swin T, and DINOv2 models under both FedAvg and FedDistill frameworks. The homogeneous preprocessing ensures that any observed performance differences arise from the model architecture or learning strategy rather than from variations in data preparation.

### 3.3 Fine-tuning of transformer architectures

The pre-trained backbones of four transformer architectures- Vision Transformer (ViT-B/16), Data-efficient Image Transformer (DeiT-Tiny), Swin Transformer (Swin-T), and DINOv2-were supplemented with unique classification heads for binary malaria detection in order to attain strong performance in both centralized and federated learning scenarios. ImageNet weights (or self-supervised weights for DINOv2) were used to start each backbone, and custom blocks tailored for medical imaging were used to fine-tune them. ViT-B/16 used 16 × 16 patch embeddings with a class token and a fine-tuning block that included GELU activations, fully linked layers (768 → 1,024 → 512 → 2), progressive dropout (0.4 → 0.3 → 0.2), and layer normalization. With class and distillation tokens, batch normalization, shaped dropout (0.6 → 0.1), and a classifier head (192 → 1,024 → 2), DeiT-Tiny used a lightweight three-layer transformer. DINOv2 used self-supervised pretraining with a classifier head (768 → 512 → 256 → 2) and dropout (0.3) for regularization, which worked especially well in federated scenarios, while Swin-T used its hierarchical patch merging and shifted window attention with a head (768 → 512 → 2) to capture local and global context.

The generalized fine-tuning block for all models can be expressed as:


$$\hat{y}=\text{Softmax}(W_{2}\;\text{ReLU}(W_{1}\;\mathrm{GAP}(F)+b_{1})+b_{2})$$ (1)

**Figure 2:**
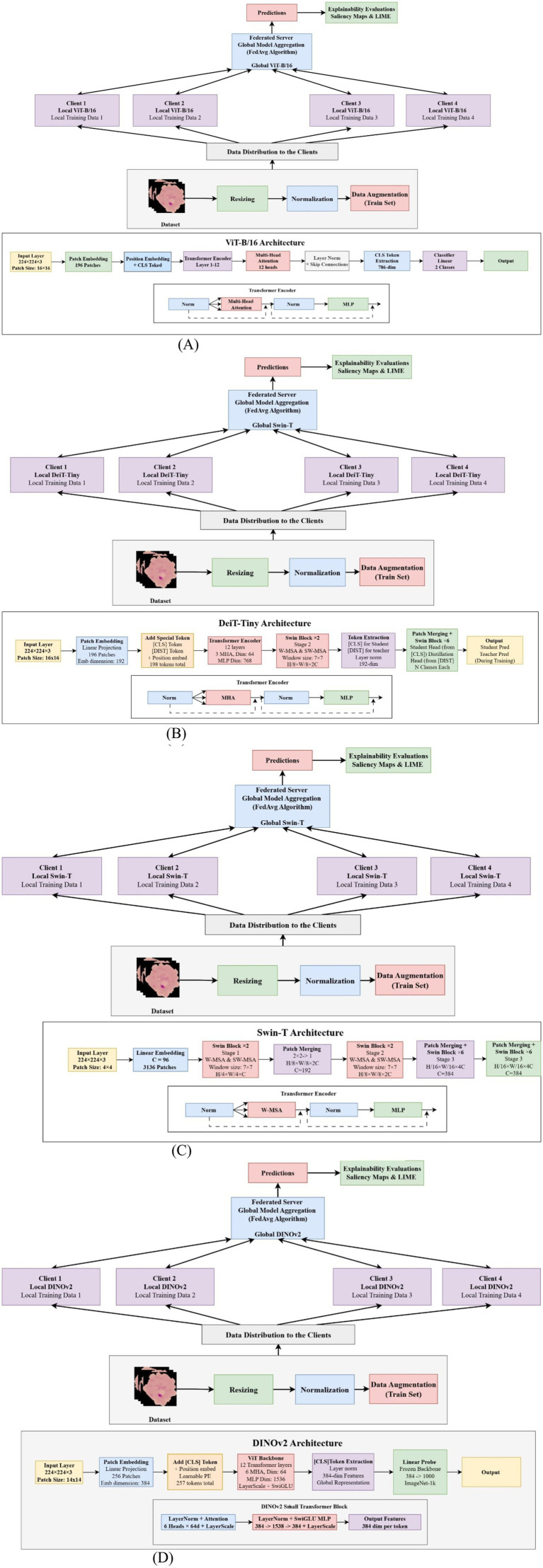
FedAvg variants Transformers architectures. **(A)** FedAvg ViT architecture. **(B)** FedAvg Diet architecture. **(C)** FedAvg SWIN architecture. **(D)** FedAvg Dino V2 architecture.

Where, F stands for extracted transformer features, GAP for global average pooling, b is the predicted probability for the binary classes (Parasitized/Uninfected), and W₁, W₂, and b₁, b₂ are learnable parameters. In class-token-based systems, the class token output is used in lieu of global average pooling. Strong performance was achieved through this customized fine-tuning strategy in both federated learning and centralized settings, demonstrating the value of specialized classification heads in advancing medical image diagnosis. Further architectural details supporting this improvement are illustrated in Figure 2, which presents the FederatedAvg variants of the transformer-based models.

In federated learning, the formal fine-tuning expression is given in Equation 1, and it is expanded in Equation 2 to include a distillation term for knowledge transmission. FedDistill uses knowledge distillation to guide local client updates, using the global model from the previous communication cycle as a teacher. The definition of the distillation loss is:


$$L_{\text{distill}}=\alpha \;L_{\mathrm{CE}}+(1-\alpha )\;T^{2}\;\mathrm{KL}(\sigma (z_{s}/T),\sigma (z_{t}/T))$$ (2)

**Figure 3:**
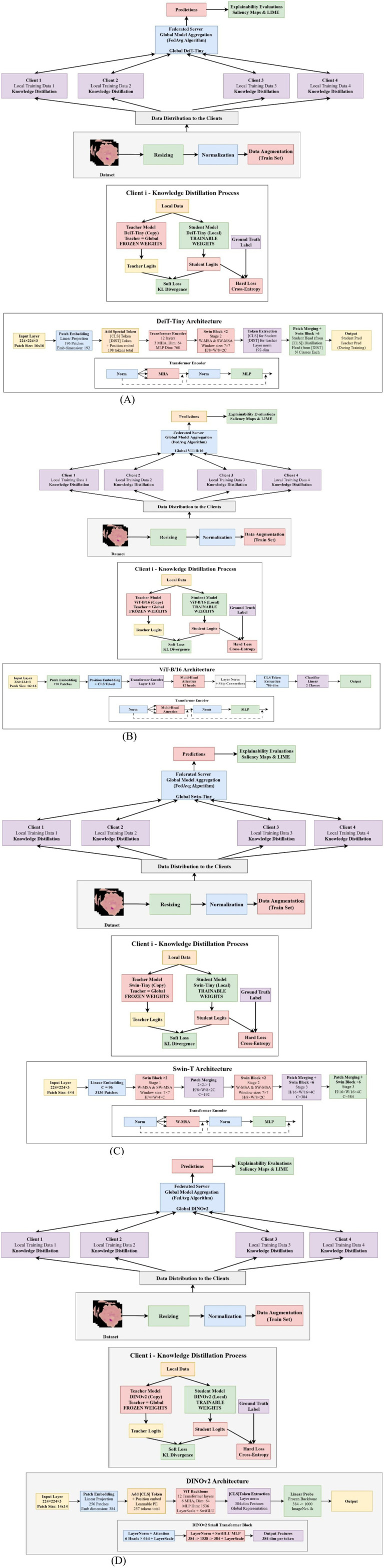
Federatedistill variants transformers architectures. **(A)** FedDistill Diet architecture. **(B)** FedDistill VIT architecture. **(C)** FedDistill SWIN architecture. **(D)** FedDistill Dino V2 architecture.

The framework combines standard cross-entropy loss with Kullback–Leibler divergence between student and teacher logits, using a distillation T = 2 and balancing both losses with α of 0.4. FedAvg models comprise approximately 85.8, 5.7, 28.2, and 22.8 million parameters, relying on periodic aggregation. FedDistill versions leverage the teacher-student paradigm to transmit knowledge efficiently during local training (33, 34). Input images are preprocessed to 224 × 224, then passed through transformer-based feature extraction, followed by global pooling or the class token mechanism, and finally refined through a tuned classification stage before aggregation or distillation. The corresponding architectural variations are illustrated in Figure 3, which presents the FederatedDistill variants of the transformer models.

#### 3.3.1 Training and hyperparameter tuning in federated malaria detection

A federated learning setting with four non-IID clients was simulated to mimic real-world healthcare heterogeneity. Four transformer variants—ViT B/16, DeiT Tiny, Swin T, and DINOv2—were trained using either Federated Averaging or Federated Distillation. Training spanned 10 global rounds, with each client performing 4 local epochs. FedDistill incorporated a knowledge distillation term with temperature two and weight alpha zero point four, while FedAvg optimized cross-entropy with label smoothing at epsilon zero point one. All models were optimized via AdamW using cosine annealing with warm restarts, gradient clipping, weight decay, and a learning rate of two times 10 raised to the negative five.

Federated Averaging (FedAvg): One of the key approaches of federated learning is Federated Averaging (FedAvg) that allows decentralized training of models on a large number of clients without sharing personal data. Each client in this model updates his local model using stochastic gradient descent (SGD) on its own private dataset. Global parameters are started on a central server.

Every client optimizes its local model $\theta_{k}^{t}\;$ through the stochastic gradient descent. On local training, clients upload updated weights $\theta_{k}^{t+1}$ mentioned to a central server, which aggregates the weights through:


$$\theta^{t+1}=\sum_{k=1}^{K}\frac{|D_{k}|}{\sum_{j}|D_{j}|}\;\theta_{k}^{t+1}$$ (3)

**Figure 4:**
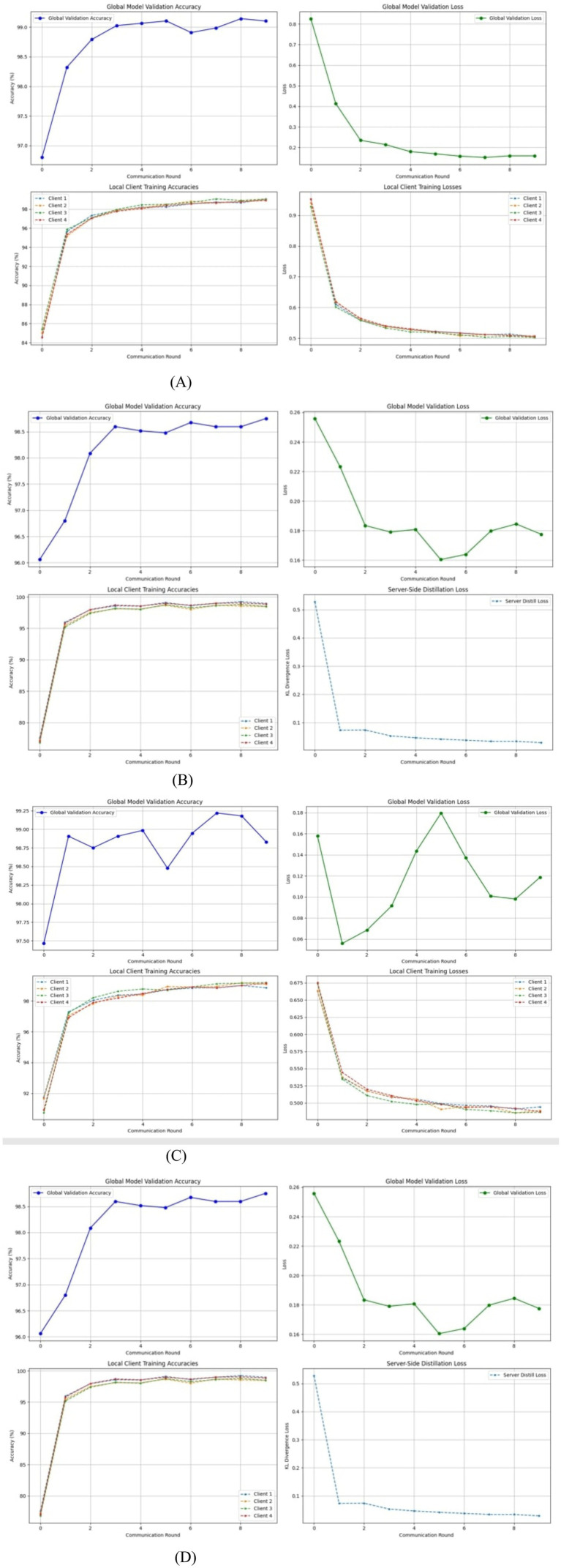
FedAvg transformers training and validation for malaria cells. **(A)** FedAvg transformers with ViT. **(B)** FedAvg transformers with DeiT-Tiny. **(C)** FedAvg transformers with Swin-T. **(D)** FedAvg transformers with DinoTransformer.

Equation 3 enforces robustness by scaling each client’s update by its data volume to ensure fair global aggregation. When the clients hold identical datasets, the method reduces to equal averaging while remaining generalizable to varied data distributions. In malaria image classification, this would allow hospitals and laboratories to train together without leaking sensitive information while adhering to privacy rules such as HIPAA and GDPR. Differences in devices, staining, and parasite prevalence create non-IID conditions; yet, FedAvg improves generalization across diverse clinical sites. Larger institutions naturally affect the global model more strongly, while smaller sites contribute useful variability. This collaborative strategy produces a reliable and widely deployable malaria detection model suited to resource-limited facilities. FedAvg was assessed using multiple transformer families, including DINOv2, Swin-T, DeiT-Tiny, and ViT B/16. The same transformer set supported the development of a comparative federated distillation framework. The training and validation behavior of the FedAvg transformer models for malaria cell analysis is illustrated in Figure 4.

#### 3.3.2 Federated distillation

It exchanges model outputs instead of weights, which improves privacy protection and drastically lowers communication cost in contrast to parameter-sharing techniques like FedAvg. Each client in this system uses its own private dataset to calculate local output logits, which are then sent to a central server. These client contributions are then averaged by the server to provide aggregated logits. Equation 4 expresses this aggregate at iteration t + 1:


$$z^{t+1}=\frac{1}{K}\sum_{k}z_{k}^{t+1}$$ (4)

The methodological advancement here lies in the refinement of the distillation process to handle high-dimensional transformer embeddings. Unlike traditional logit-based distillation, our framework utilizes a teacher-student-global paradigm where the teacher model is a frozen snapshot of the converged global state from the prior round, forcing the student model to align its global attention maps with the global consensus while optimizing local discriminative features.

Where, N represents the total number of samples across all clients.

With a temperature scaling factor T to soften the distributions, the server uses these aggregated logits to enhance the global model by reducing the Kullback–Leibler (KL) divergence between its predictions and the aggregated outputs. The following is the definition of the associated knowledge distillation loss, as expressed in Equation 5:


$$L_{\mathrm{KD}}=T^{2}\;\mathrm{KL}(\sigma (z_{\text{global}}/T)\|\;\sigma (z^{\left(t+1\right)}/T))$$ (5)

A more private and effective collaborative training framework is made possible by the server integrating the pooled expertise of all clients via this distillation process, which does not need direct access to raw data or local model parameters.

**Figure 5:**
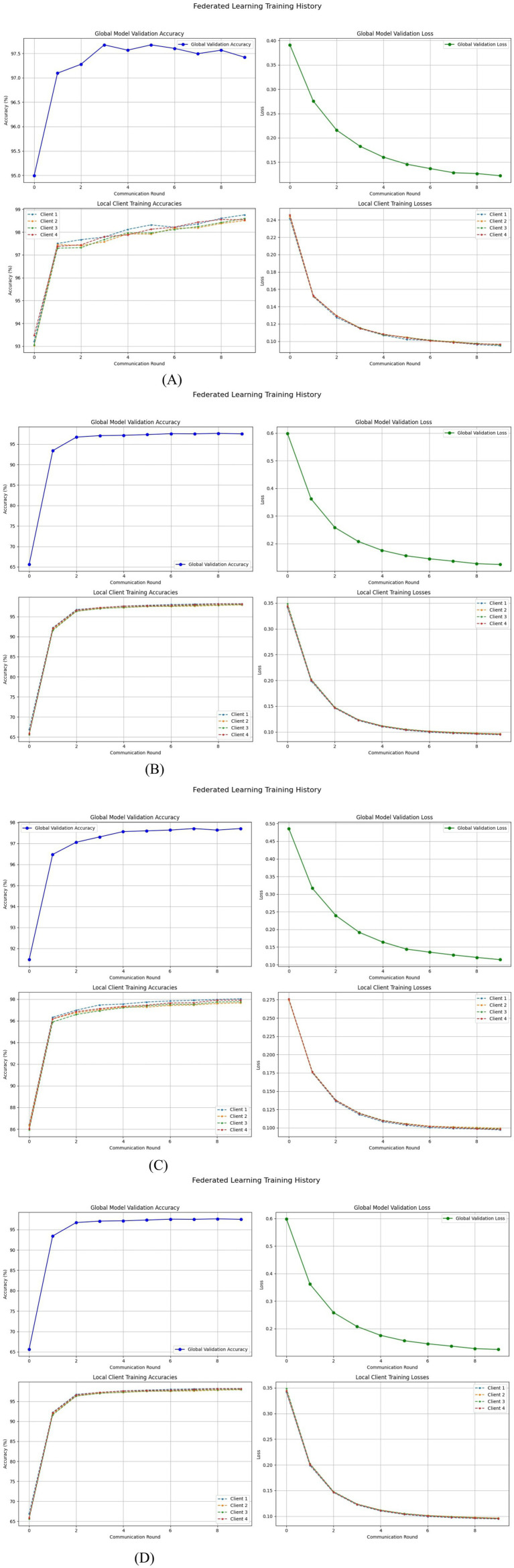
FedDistill transformers training and validation for malaria cells. **(A)** FedDistill Transformers with ViT. **(B)** FedDistill Transformers with DeiT-Tiny. **(C)** FedDistill Transformers with Swin-T. **(D)** FedDistill Transformers with Dinov2.

Student–teacher–global model dynamics: FedDistill innovatively combines a student-teacher paradigm into federated learning, allowing local adaptation while maintaining global consistency. Each client will train a student model on local data, guided by a fixed teacher initialized from the previous global model, avoiding overfitting to local peculiarities. In each round, the server shares the global model, which initializes both the student and teacher models, and aggregates the updates from the students to form the next global model. Such an approach achieves a good balance in heterogeneous clients, preserves privacy, and is well-suited to healthcare situations in which clients have some bandwidth or legal limitations. Employing compact logits and transformer architectures including DINOv2, Swin, DeiT-Tiny, and ViT B/16 enables accurate malaria classification even in resource-limited regions. The training and validation performance of the FedDistill transformer models for malaria cell detection is presented in Figure 5.

Transformer and CNN model architectures: Every one of the three transformer models that have already been trained and enhanced with a distinct classification head for the detection of malaria is evaluated:

The Vision Transformer, consisting of 85.8 million parameters, processes input images by first dividing them into 16 × 16 patches, resulting in 196 tokens. These tokens are then passed through a 3-layer MLP head with dimensions 768 → 1,024 → 512 → 2, followed by 12 transformer layers with 12 attention heads. The architecture also incorporates LayerNorm, GELU activation, and dropout with progressively decreasing rates of 0.4, 0.3, and 0.2.

The DeiT-Tiny model, with 5.7 million parameters, employs three transformer layers with three attention heads and uses 192-dimensional embeddings. Its deep classifier head increases complexity from 192 to 1,024 before reducing it to 2, incorporating GELU activation, BatchNorm, and shaped dropout that decreases progressively from 0.6 to 0.1.

The Swin Transformer has 28.29 million parameters and is hierarchically designed with patch merging and shifted-window self-attention to gradually increase the features from 96 to 768. Its classifier head follows a similar structure to that of ViT with a dimensionality of 768 to 512 to 2, enabling the model to generate rich representations that capture both local and global contexts.

DINOv2 (dinov2-small-imagenet1k-1-layer) has 22.8 million parameters and is based on the Vision Transformer architecture for self-supervised learning of robust visual features. The model leverages its pre-trained capabilities for generic feature extraction and fine-tunes them for the malaria detection task with a patch size of 14 by 14, incorporating a classification head with a structure of 768 to 512 to 256 to 2 along with LayerNorm and a dropout rate of 0.3.

Training and optimization: Every setup makes use of the same optimization pipelines:

- Optimizer: AdamW (*β*₁ = 0.9, β₂ = 0.999)
- Learning Rate: 2 × 10^−5^ with cosine annealing and warm restarts (T₀ = 10, T_mlt_ = 2, η_m_ᵢ_n_ = 10^−6^)
- Weight Decay: 1 × 10^−4^
- Loss: Cross-entropy with label smoothing (*ε* = 0.1)
- Gradient Clipping: Max norm = 1.0
- Callbacks: ReduceLROnPlateau (patience = 7) and EarlyStopping (patience = 10)

Hyperparameter sweeps over batch size, dropout scheduling, learning rate, and training rounds identified four local epochs and four global communication rounds as the optimal configuration for federated learning frameworks. Fixed random seeds ensured reproducibility, and each setup was run three times to reduce single-run bias, with results reported as averages with standard deviations. Accuracy fluctuations remained below 0.3%, confirming reliability without overfitting. Robustness tests included learning rates of one e minus five, two e minus five, and five e minus five, batch size of 32, and varied dropout rates. Improvements were restricted to the training sets to prevent data leakage and maintain the integrity of the test and validation sets.

Several CNN designs and their ensembles were assessed for comparison: VGG16, with 138.3 million parameters, follows its standard architecture of 13 convolutional layers and three fully connected layers. For the malaria detection task, the final layer was modified to include a dropout rate of 0.5 and adapted for binary classification with 2 output units.

ResNet50, with 25.5 million parameters, employs a standard 50-layer residual network architecture with batch normalization. For the malaria detection task, the final fully connected layer of the model was modified to produce a binary output.

EfficientNetB3, which has 12.2 million parameters, applies compound scaling to optimize depth, width, and resolution. For the malaria detection task, its final layer was modified to perform binary classification.

The ResNet50 + VGG16 Ensemble, with approximately 163.6 million parameters, leverages complementary feature representations from both architectures. The ResNet50 plus VGG16 ensemble, with approximately 163.6 million parameters, leverages complementary feature representations from both architectures. Predictions from the two models are combined using weighted averaging to enhance overall performance. The performance of the ResNet50 plus VGG16 ensemble and the performance of individual CNN models along with their ensemble is summarized in Table 1.

**Table 1:** Hyperparameters and comparative performance of vision transformer and cnn-based models.

Model	Learning rate	Batch size	Dropout	Epochs	Accuracy (%)	MCC	Cohen’s Kappa	Precision (%)	Recall (%)	F1-score (%)
ViT-B/16	2 × 10^−5^	32	[0.4, 0.3, 0.2]	10/10	96.88	0.9375	0.9375	96.9	96.9	96.9
DeiT-Tiny	2 × 10^−5^	32	[0.6 → 0.1]	10/10	96.91	0.9383	0.9383	96.9	96.9	96.9
Swin-T	2 × 10^−5^	32	[0.4, 0.3, 0.2]	10/10	97.75	0.9550	0.9550	97.8	97.8	97.8
DINOv2	2 × 10^−5^	32	Default	10/10	97.22	0.9444	0.9444	97.2	97.2	97.2
VGG16	2 × 10^−5^	32	0.3	20/20	97.61	0.9522	0.9522	97.6	97.6	97.6
ResNet50	2 × 10^−5^	32	0.3	20/20	97.61	0.9522	0.9522	97.6	97.6	97.6
EfficientNetB3	2 × 10^−5^	32	0.3	20/20	97.61	0.9522	0.9522	97.6	97.6	97.6
ResNet50 + VGG16 Ensemble	2 × 10^−5^	32	0.3	20/20	98.15	0.9630	0.9630	98.2	98.2	98.2

Every model used the same optimization pipeline:

- Optimizer: AdamW (*β*₁ = 0.9, β₂ = 0.999)
- Learning Rate: 2 × 10^−5^ with cosine annealing and warm restarts (T₀ = 10, T_mult = 2, *η* min = 10^−6^)
- Weight Decay: 1 × 10^−4^
- Loss: Cross-entropy with label smoothing (*ε* = 0.1)
- Gradient Clipping: Max norm = 1.0
- Callbacks: ReduceLROnPlateau (patience = 7) and EarlyStopping (patience = 10)

#### 3.3.3 Transformer architectures for malaria detection

The transformer-based designs DINOv2, Swin Transformer (Swin-T), Data-efficient Image Transformer (DeiT-Tiny), and Vision Transformer (ViT-B/16) are used to diagnose malaria. In order to extract hierarchical representations from medical pictures, each model provides a unique method.

Through the division of an input picture into a series of non-overlapping patches, the ViT-B/16 model presents a paradigm shift. These patches are processed using stacked transformer encoder blocks that use multi-head self-attention after being linearly inserted into tokens. In order to collect global contextual information throughout the whole picture, the resultant feature vectors are aggregated using global average pooling (GAP) and then categorized using a fully connected (FC) layer and a softmax function. This extraction and classification pipeline is formalized in Equation 6:


Output=Softmax(FC(GAP(Transformer(X)))) (6)

Where, X represents the input image.

The DeiT-Tiny architecture extends this framework, and is well suited in less data-intensive situations because it also includes a distillation token to facilitate the exchange of knowledge between students and teachers during training. Equation 7 illustrates how its learning goal balances a distillation loss and cross-entropy loss using a weighting factor *λ*.


$$L_{\text{total}}=\lambda \cdot L_{\mathrm{CE}}+(1-\lambda )\cdot T^{2}\cdot \mathrm{KL}(\begin{array}{l} \text{Softmax}(y_{\text{student}}/T)\;\\ \text{Softmax}(y_{\text{teacher}}/T) \end{array})$$ (7)

Where, T is the temperature scaling parameter.

Having a hierarchical structure with pictures being partitioned into moving windows, Swin-T builds on these concepts and allows localized self-attention to be computed efficiently with maintaining global dependence. Similar to ViT, Swin-T operates with GAP to extract hierarchical features, which are subsequently divided by classification by mapping them through an FC-softmax layer, as shown in Equation 8:


Output=Softmax(FC(GAP(SwinTransformer(X)))) (8)

DINOv2 generates reliable and broadly applicable visual representations by using self-supervised pre-training on extensive picture datasets. Models may acquire strong features without centralizing sensitive data by adapting its student-teacher distillation architecture, which incorporates centering and sharpening, to federated learning.

Federated Distillation (FedDistill) and Federated Averaging (FedAvg) are two federated learning frameworks that include these transformer topologies to provide privacy-preserving training. A central server uses a weighted average based on dataset sizes to aggregate model weights from N dispersed clients in the FedAvg setup, as given in Equation 9:


$$w^{\left(t+1\right)}=\frac{1}{N}\sum_{n=1}^{N}w_{n}^{\left(t\right)}$$ (9)

Where, $w_{n}^{\left(t\right)}$ denotes the parameters from client n at communication round t, |D_n_| is the client dataset size, and |D| is the total data across all clients.

A knowledge distillation mechanism is added by FedDistill to this approach: local clients train student models, when the server transforms their combined knowledge- represented by softened logits- into an updated global model. In decentralized, privacy-sensitive settings, the teacher-student paradigm guarantees efficient malaria diagnosis and improves representational generalization.

### 3.4 Classification pipeline and output generation

The final classification framework for transformer-based malaria detection models leverages hierarchical feature representations from the backbone while maintaining computational efficiency. Each model follows a standardized pipeline consisting of a transformer backbone, a dropout regularization layer, and a fully connected classification layer. Input images of 224 × 224 pixels are processed by the backbone to generate high-dimensional embeddings, with global representations captured through distillation tokens in DeiT or class tokens in ViT and Swin Transformer. Dropout mitigates overfitting, and the embeddings are mapped through the fully connected layer to produce a two-class probability distribution normalized using the softmax activation function represented in Equation 10.


$$P_{\text{predicted}}=\mathrm{SAF}(\mathrm{FC}(\text{Dropout}(c)))$$ (10)

Where, c denotes the token embedding from the backbone. The softmax activation function with optional temperature scaling T is defined in Equation 11:


$$\mathrm{SAF}(y_{i})=\frac{\exp(\frac{y_{i}}{T})}{\sum_{j=1}^{C}\exp(\frac{y_{j}}{T})},\;\;C=2$$ (11)

In distillation applications, where it softens probability distributions for information transmission, the temperature parameter T is very important. While enabling the classification head to transform learnt embeddings into task-specific probabilistic predictions, the method guarantees resilience against overfitting.

Different optimization algorithms are used for FedAvg along with FedDistill frameworks in federated learning adaptations. FedAvg models use global weighted averaging of model parameters to synchronize after training locally on each client. In order to enhance generalization, FedDistill models use a teacher-student paradigm and a hybrid loss function that combines knowledge distillation (KD), which is specified by Equation 12, with cross-entropy (CE).


$$\begin{array}{l} L_{\text{total}}=\alpha \;L_{\mathrm{CE}}(y_{\text{pred}},y_{\text{true}})+(1-\alpha )\\ \;T^{2}\;\mathrm{KL}(\mathrm{SAF}(\frac{y_{\text{student}}}{T})\;\;\mathrm{SAF}(\frac{y_{\text{teacher}}}{T})) \end{array}$$ (12)

In this case, T smoothes the probability for stable distillation, while α = 0.4 balances the contributions of the CE and KD factors. In the FedDistill configuration, this framework was applied to all transformer designs, including FedDistill-ViT-B/16, FedDistill-DeiT-Tiny, FedDistill-Swin-T, and FedDistill-DINOv2.

In general, the procedure combines softmax-based classification, dropout regularization, transformer-based feature extraction, and image preprocessing. Further federated solutions that provide efficient distributed learning while protecting data privacy across participating healthcare facilities include teacher-guided knowledge distillation (FedDistill) and global weight aggregation (FedAvg).

### 3.5 Federated learning strategy for transformer-driven malaria detection

Sensitive medical information is kept localized among dispersed clients by using a federated learning (FL) framework to provide privacy-preserving collaborative training of sophisticated transformer-based architectures for malaria diagnosis. The paradigm evaluates eight model variations across the FedDistill and FedAvg frameworks. A client–server protocol iteratively synchronizes parameters, initialized via ImageNet or self-supervised pre-training with DINOv2, to optimize empirical risk across decentralized private datasets. It is important to note that while standard FedAvg shares model gradients, which can be susceptible to reconstruction attacks, the FedDistill approach enhances privacy by sharing only soft predictions, or logits, on a public anchor dataset. This gradient-free communication prevents direct leakage of model parameters and significantly reduces the attack surface for model inversion attacks.

The global model parameters $\;w^{\left(0\right)}$ , which were acquired via ImageNet pre-training or, in the case of DINOv2, self-supervised pre-training, are initialized at the start of the training process. All N clients get the global parameters t from the server during communication round $w^{\left(t\right)}$ . The empirical risk on each client’s private dataset is then optimized using Equation 13:


$$\min_{w}\;{ℒ}_{\text{local}}^{\left(k\right)}(w)=\frac{1}{|D_{k}|}\sum_{(x_{i},y_{i})\in D_{k}}\ell (f(x_{i};w),y_{i}),$$ (13)

In this case, FedAvg models instantiate the classification loss function l as cross-entropy, while FedDistill models use a combined knowledge distillation loss. The server uses weighted federated averaging to aggregate the updated parameters $w_{k}^{\left(t+1\right)}$ that clients transmit back when local training is finished, guaranteeing proportionate contributions depending on dataset size, as defined in Equation 14:


$$w^{\left(t+1\right)}=\sum_{k=1}^{N}\frac{|D_{k}|}{\sum_{j=1}^{N}|D_{j}|}\cdot w_{k}^{\left(t+1\right)}$$ (14)

FedDistill models use an extra teacher-student process in which the prior global model is treated as a teacher by each client. Kullback–Leibler (KL) divergence and cross-entropy (CE) loss are used in the local training goal to create a convex combination, shown in Equation 15:


$$\begin{array}{l} L_{\text{distill}}=\alpha \cdot L_{\mathrm{CE}}+(1-\alpha )\cdot T^{2}\cdot \mathrm{KL}\\ \left(\text{softmax}(\frac{z_{\text{student}}}{T})\;\;\text{softmax}(\frac{z_{\text{teacher}}}{T})\right) \end{array}$$ (15)

The probability distributions are smoothed by T=2 , and the CE and knowledge distillation factors are balanced by α=0.4 .

The AdamW optimizer, batch size, learning rate $\mathrm{LR}=2\times 10^{-5}$ , local epochs E=5 , and communication rounds R=10 are the hyperparameters that are kept constant throughout all model variations. Equation 16 represents the total federated update for FedDistill models:


$$w^{\left(t+1\right)}=\text{FedAvg}({\left\{w_{k}^{\left(t+1\right)}\right\}}_{k=1}^{N}),\;\;\min_{w}L_{\text{distill}}(w,w_{\text{teacher}})$$ (16)

A framework for malaria diagnosis that is accurate, scalable, and privacy-preserving is offered by this federated learning approach. Strong performance metrics are shown in the next sections, confirming the efficacy of this technology and emphasizing its potential for expansion into more general medical imaging applications.

### 3.6 Hardware and software setup

Experiments were performed in the high-performance computer environment to ensure the transformer-based deep learning models would be repeatable and have the highest possible effect. The implementations were based on PyTorch and the Hugging Face Transformers package and supported by dedicated Python scripts to do the data pretreatment, augmentation and analysis.

The hardware consisted of Intel Xeon Gold 6248R at 3.00 GHz, 128 GB of DDR4 RAM, and storage of 2 TB of NVMe SSD and NVIDIA Tesla P100 graphics that were donated through the Kaggle. The runtime was Ubuntu 22.04 LTS, and optimized computational performance, particularly in federated learning simulations, was achieved with mixed-precision training and data-parallelism.

The federated learning system was simulated in one high-performance work station to provide a controlled experiment and repeatability. A real multi-institutional deployment is not fully simulated despite the simplification of effective assessment provided by this approach.

## 4 Results and analysis

In this section, a comprehensive study of transformer-based malaria detection systems under centralized and federated learning models is discussed. They have compared 16 model configurations, 8 federated and 8 centralized, such as Federated Distillation (FedDistill) and Federated Averaging (FedAvg)-based implementations. In order to provide a robust determination of the effectiveness, the performance of this model was evaluated based on a set of measures which included accuracy, ROC-AUC, Matthews correlation coefficient (MCC), Cohen Kappa as well as parameter efficiency.

### 4.1 Performance indicators

A large variety of statistical indicators were employed to give an in-depth assessment of the categorization models. Accuracy is defined in Equation 17 as a %age of properly identified samples.


$$\text{Accuracy}=\frac{\mathrm{TP}+\mathrm{TN}}{\mathrm{TP}+\mathrm{TN}+\mathrm{FP}+\mathrm{FN}}$$ (17)

In the scenario, FP and FN will be false positives and false negatives, respectively, and TP and TN will denote true positives and true negatives. Considering all of the four categories of confusion matrices, the Matthews Correlation Coefficient (MCC), as presented in Equation 18 provides an objective measurement of the quality of prediction:


$$\mathrm{MCC}=\frac{\left(\mathrm{TP}\times \mathrm{TN})-(\mathrm{FP}\times \mathrm{FN}\right)}{\sqrt{\left(\mathrm{TP}+\mathrm{FP})(\mathrm{TP}+\mathrm{FN})(\mathrm{TN}+\mathrm{FP})(\mathrm{TN}+\mathrm{FN}\right)}}$$ (18)

Specificity and accuracy are parameterized by Equation 19 as the measure of the ability to have an accurate detectability of negative and positive occurrences respectively:


$$\text{Specificity}=\frac{\mathrm{TN}}{\mathrm{TN}+\mathrm{FP}},\;\;\text{Precision}=\frac{\mathrm{TP}}{\mathrm{TP}+\mathrm{FP}}$$ (19)

The inter-rater agreement formula (Cohen Kappa) in Equation 20 where the agreement that is attributed to chance is corrected is:


$${\text{Cohen}}^{’}s\;\text{Kappa}=\frac{\text{observed value}-\text{expected value}}{1-\text{expected value}}$$ (20)

Equation 21 expresses the F1-score, which combines accuracy and recall into a single metric:


$$F1-\text{score}=2\times \frac{P\times R}{P+R}$$ (21)

Furthermore, ROC-AUC provided a threshold-independent measure of model discrimination by quantifying the trade-off between true positive rate (TPR) with false positive rate (FPR) across thresholds. Scikit-learn was used to calculate all measures in order to guarantee repeatability and consistency between tests.

### 4.2 Evaluation results under federated learning

All transformer models achieved excellent global performance under federated learning. FedAvg-ViT-B/16 reached 96.88% accuracy, while FedAvg-DeiT-Tiny and FedAvg-Swin-T achieved 96.91 and 97.75%, respectively. FedAvg-DINOv2 attained 97.22%, matching centralized results. FedDistill-DeiT-Tiny achieved the highest 97.79% accuracy, while FedDistill-DINOv2 reached 95.81%, balancing accuracy and efficiency. Client accuracies ranged from 97.02 to 98.98%. Hyperparameter analysis identified optimal stability at a temperature of 5 and a weighting factor of 0.5, ensuring effective knowledge distillation and model convergence. The detailed performance of the FedAvg models and the performance of the FedDistill models is presented in Table 2.

**Table 2:** Comparative performance metrics of FedAvg and FedDistill models.

Model	Global accuracy (%)	ROC-AUC	MCC	Cohen’s Kappa	Best client accuracy (%)
FedAvg-ViT-B/16	96.88	0.9940	0.9375	0.9375	98.98
FedAvg-DeiT-Tiny	96.91	0.9932	0.9383	0.9383	97.02
FedAvg-Swin-T	97.75	0.9968	0.9550	0.9550	98.84
FedAvg-DINOv2	97.22	0.9722	0.9444	0.9444	98.15
Performance of FedDistill models					
FedDistill-ViT-B/16	97.45	0.9958	0.9490	0.9490	98.25
FedDistill-DeiT-Tiny	97.79	0.9966	0.9557	0.9557	98.67
FedDistill-Swin-T	97.62	0.9962	0.9524	0.9524	98.42
FedDistill-DINOv2	95.81	0.9581	0.9162	0.9162	96.75

### 4.3 Comprehensive classification reports

For categorization, the best-performing federated learning models did extremely well in both classes. Considering the limitations imposed by privacy preservation in federated learning, the performance of FederatedDistill-DeiT-Tiny and FederatedAvg-Swin-T was very strong, with no class bias, achieving near-perfect accuracy, recall, and F1-scores of 0.98 for both parasitized and uninfected classes. The per-class metrics of these best-performing models are detailed in Table 3.

**Table 3:** Per-class metrics of best models.

Model	Class	Precision	Recall	F1-score	MCC	Cohen’s Kappa
FederatedDistill-DeiT	Parasitized	0.98	0.98	0.98	0.9557	0.9557
	Uninfected	0.98	0.98	0.98	0.9557	0.9557
FederatedAvg-Swin-T	Parasitized	0.98	0.98	0.98	0.9550	0.9550
	Uninfected	0.98	0.98	0.98	0.9550	0.9550
FedAvg-DINOv2	Parasitized	0.9731	0.9698	0.9715	0.9444	0.9444
	Uninfected	0.9713	0.9745	0.9729	0.9444	0.9444
FedDistill-DINOv2	Parasitized	0.9652	0.9485	0.9568	0.9162	0.9162
	Uninfected	0.9516	0.9674	0.9594	0.9162	0.9162

FederatedDistill-DeiT-Tiny and FederatedAvg-Swin-T showed an excellent balance, with Cohen’s Kappa and MCC values of 0.9557 and 0.9550, respectively. FedAvg-DINOv2 and FedDistill-DINOv2 also showed the MCC of 0.9444 and 0.9162, respectively, confirming reliable classification. Overall, strong agreement across the metrics reflects the capacity of both frameworks to learn effectively from decentralized data while preserving high diagnostic accuracy, which is necessary for clinical applications.

### 4.4 Client-based performance assessment

Client-level evaluation manifested that both federated learning frameworks had steady convergence tendencies across all communication cycles. FederatedDistill-DeiT-Tiny had a consistent progress, with the client accuracies ranging from 97.88 to 98.43%, and the associated losses fell from 0.1082 to 0.0985 over 10 rounds. The performance of FederatedAvg-Swin-T showed dependable local optimization as well; its losses decreased from 0.2381 to 0.2268, while client accuracies for this model were between 98.02 and 98.57%. All clients demonstrated strong agreement metrics with Cohen’s Kappa scores ranging from 0.953 to 0.957 and MCC values between 0.954 and 0.958, indicating very well-balanced performances. The individual client performance of the best models is summarized in Table 4.

**Table 4:** Individual client performance of best models.

Model	Client	Training accuracy (%)	Loss	MCC	Cohen’s Kappa	Communication rounds
FederatedDistill-DeiT	Client 1	97.88	0.1082	0.954	0.953	10
	Client 2	98.12	0.1039	0.956	0.956	10
	Client 3	98.22	0.1007	0.957	0.957	10
	Client 4	98.43	0.0985	0.958	0.957	10
FederatedAvg-Swin-T	Client 1	98.02	0.2381	0.955	0.954	10
	Client 2	98.28	0.2331	0.956	0.956	10
	Client 3	98.42	0.2291	0.957	0.957	10
	Client 4	98.57	0.2268	0.958	0.958	10

The high Cohen’s Kappa scores, ranging from 0.953 to 0.958 and MCC values between 0.954 and 0.958, signify a strong agreement between the true and predicted labels. Besides, the small client variation of 0.004 or less shows that the federated learning framework effectively handles diverse datasets with no loss in diagnostic accuracy. This depicts consistent learning across clients, stability across classes, and real-world clinical robustness. These strong agreement metrics confirm the effectiveness of FedAvg and FedDistill in enabling high-quality, decentralized model training for medical image diagnosis so as to ensure both reliability and precision across distributed healthcare environments.

### 4.5 Statistical verification and cross-dataset performance

To validate the statistical significance of the proposed models and ensure the results are not due to random chance, we performed the Wilcoxon Signed-Rank Test. As shown in Table 5, all federated models demonstrated statistically significant performance with *p*-values < 0.001 at a significance level of *α* = 0.05. The introduction of the NM-AIST dataset mimics a real-world deployment where hospitals use different imaging hardware. Despite this acquisition heterogeneity, the FedDistill-Swin-T model maintained a high precision of 92.55% and an MCC of 0.8461, demonstrating that the knowledge distillation process effectively mitigates the performance drop usually associated with cross-institutional domain shifts.

**Table 5:** Statistical verification results using the Wilcoxon signed rank test.

Model	Wilcoxon statistic	*p*-value	Significance (α = 0.05)
FedAvg-ViT-B/16	0.0000	0.0010	Significant
FedAvg-DeiT-Tiny	0.0000	0.0010	Significant
FedAvg-Swin-T	0.0000	0.0010	Significant
FedAvg-DINOv2	0.0000	0.0010	Significant
FedDistill-ViT-B/16	0.0000	0.0010	Significant
FedDistill-DeiT-Tiny	0.0000	0.0010	Significant
FedDistill-Swin-T	0.0000	0.0010	Significant
FedDistill-DINOv2	0.0000	0.0010	Significant

Furthermore, the performance of the models on the heterogeneous NM-AIST dataset is summarized in Table 6. The FedAvg-ViT-B/16 achieved an accuracy of 92.89% and FedDistill-Swin-T achieved 92.09%, demonstrating robust generalization across different data sources.

**Table 6:** Comparative performance metrics of FedAvg and FedDistill models.

Model	Accuracy (%)	Precision (%)	Recall (%)	F1-score (%)	MCC	Cohen’s Kappa
FedAvg models						
FedAvg-ViT-B/16	92.89	92.89	92.89	92.89	0.8577	0.8577
FedAvg-DeiT-Tiny	92.28	92.46	92.28	92.26	0.8472	0.8452
FedAvg-Swin-T	92.49	92.69	92.49	92.48	0.8517	0.8496
FedAvg-DINOv2	91.38	91.40	91.38	91.38	0.8277	0.8275
FedDistill models						
FedDistill-ViT-B/16	91.40	91.60	91.40	91.38	0.8298	0.8276
FedDistill-DeiT-Tiny	91.74	91.84	91.74	91.73	0.8357	0.8346
FedDistill-Swin-T	92.09	92.55	92.09	92.06	0.8461	0.8413
FedDistill-DINOv2	90.47	90.48	90.47	90.47	0.8094	0.8094

### 4.6 Ablation study

To validate the efficacy of the different components that the propose in the FedMal-XAI framework, performed an ablation study on the FedDistill-DeiT-Tiny model. The examined three aspects: the effect of the distillation weight (α), the scaling parameter of the temperature (T), and the effect of the pipeline of data augmentation.

#### 4.6.1 Impact of distillation components

The FedDistill loss function is built as a weighted sum of Cross-Entropy loss and Kullback–Leibler divergence, where the weighting coefficient is denoted as α. As shown in Equation 12, when α is set to 1.0, the distillation component is completely removed and the procedure reduces to standard local training. Table 7 shows that the highest classification accuracy of 97.79% is achieved using the hybrid mechanism with α equal to 0.4. This performance exceeds the accuracy obtained using the Cross-Entropy-only configuration with α equal to 1.0, which reaches 96.95%, and the distillation-only configuration with α equal to 0.0, which reaches 95.40%. These results support the importance of the teacher–student signal for improving generalization across non-IID clients. Furthermore, we investigated the temperature parameter T, which softens the probability distributions, and found that a temperature value of 2 provides the best trade-off for preserving structural information between the teacher and student models.

**Table 7:** Ablation study of FedDistill-DeiT-Tiny components.

Configuration	Variation	Accuracy (%)	ROC-AUC
Distillation Weight (α)	α = 1.0 (No KD / Pure CE)	96.95	0.9810
	α = 0.0 (Pure KD)	95.40	0.9755
	α = 0.4 (Proposed)	97.79	0.9966
Temperature (T)	T = 1 (No Softening)	97.12	0.9880
	T = 5	97.45	0.9910
	T = 2 (Proposed)	97.79	0.9966
Data Processing	No Augmentation	95.39	0.9720
	With Augmentation	97.79	0.9966

#### 4.6.2 Impact of data augmentation

Given the limited data available per client in a federated setting, we employed an aggressive augmentation pipeline. Removing these augmentations resulted in a significant performance drop of 2.4%, confirming that geometric and color transformations are essential for preventing overfitting in transformer-based malaria detection, as shown in Table 7.

## 5 Model explainability

Federated transformer architectures were integrated with an explainable AI framework to improve interpretability in malaria parasite detection from blood smear images. Two federated paradigms, including Federated Averaging and Federated Distillation, were conducted on Vision Transformer Base-16, DeiT-Tiny, Swin-T, and DINOv2 using the NIH Malaria dataset. Interpretability was guaranteed via Saliency Maps and Local Interpretable Model-Agnostic Explanations, showing transparency under the non-IID setting. Quantitative analysis provided an average fidelity of 0.84 and robustness of 0.81, confirming the reliability of the explanation. To rigorously evaluate interpretability, we employed the Prediction Faithfulness (Fidelity) metric. Fidelity measures the drop in probability when salient features are occluded. It is formally defined in Equation 22 as:


$$\text{Fidelity}(x)=E_{x}[\|f(x)-f(x⊙(1-M_{S}))\|]$$ (22)

Where *f(x)* is the model’s prediction on the original image, and *M_S_* is the binary mask derived from the saliency map S. A higher score indicates that the explanation accurately identifies features crucial for the model’s decision.

These results ensured consistent interpretability and trustworthy visual reasoning, crucial for clinical deployment in decentralized and privacy-sensitive medical environments.

ViT-B/16 divides input pictures $x\in {\mathbb{R}}^{H\times W\times C}$ into $N=\mathrm{HW}/P^{2}$ patches of fixed size $x_{p}\in {\mathbb{R}}^{N\times (P^{2}\cdot C)}$ , where P is the patch dimension. L stacked transformer encoder layers handle these patches once they have been linearly embedded and merged with learnable positional encodings. Every layer incorporates residual connections, normalization, and multi-head self-attention (MSA). The definition of multi-head self-attention is provided in Equation 23:


$$\text{Attention}(Q,K,V)=\text{softmax}(\frac{{\mathrm{QK}}^{T}}{\sqrt{d_{k}}})V,$$ (23)

Where, Q,K,V stands for query, key, and value projections, and $d_{k}$ is the key dimension. The fine-grained contextual modeling made possible by this global attention process is essential for spotting minute parasite characteristics in erythrocytes.

In order to enable effective student-teacher training on smaller datasets, DeiT-Tiny expands on ViT by adding a distillation token in addition to the class token. This solution is perfect for diverse federated contexts with fluctuating resources since it maximizes computing economy while maintaining ViT’s representational capability.

Swin-T uses shifted-window attention and a hierarchical design. In order to facilitate cross-window interactions, attention is calculated inside non-overlapping windows of size M×M and moved by M/2 in succeeding layers. This method facilitates effective high-resolution malaria picture processing by lowering computing complexity from $O(N^{2})$ to O(N) .

DINOv2 uses strong visual feature extraction through self-supervised learning. The model can learn discriminative representations while retaining interpretability thanks to its knowledge distillation methodology, which has been modified for federated environments.

Weighted averaging is used in FedAvg to update the global model parameters at communication round t + 1 using Equation 24:


$$w_{t+1}=\sum_{k=1}^{K}\frac{n_{k}}{n}w_{t+1}^{k},$$ (24)

Where, $w_{t+1}^{k}$ are client-updated parameters, $n_{k}$ is the local dataset size, and $n=\sum n_{k}$ . The impact of non-IID data distributions among dispersed institutions is lessened by this aggregate.

FedDistill gives customers the ability to distill soft logits into a global student model and train local models. The loss of knowledge distillation is described in Equation 25 as follows:


$$L_{\mathrm{KD}}=\alpha \cdot L_{\mathrm{CE}}(S,y)+(1-\alpha )\cdot T^{2}\cdot \mathrm{KL}(\frac{T}{\tau }∥\frac{S}{\tau })$$ (25)

Where, as α balances hard and soft supervision, τ is the temperature for probability softening, $L_{\mathrm{CE}}$ represents cross-entropy loss, and KL stands for Kullback–Leibler divergence. Under stringent privacy and communication restrictions, this method improves generalization.

Saliency Maps backpropagate output scores in relation to input pictures in order to calculate gradient-based pixel-level attributions. In order to provide instance-level interpretability of intricate transformer choices, LIME fits interpretable local surrogate models. When combined, these XAI methods turn federated transformers from opaque black-box models into frameworks that are transparent and clinically interpretable, making them appropriate for use in diagnostic applications.

### 5.1 Model attention via saliency maps

Saliency maps, which offer gradient-based feature significance visualizations, were utilized in our research to precisely pinpoint parasite signals inside erythrocyte pictures (35). Equation 26 defines the saliency score S , which measures each input pixel x ‘s gradient-based contribution to the projected class $y_{c}$ :


$$S=|\frac{\partial y_{c}}{\partial x}|$$ (26)

**Figure 6:**
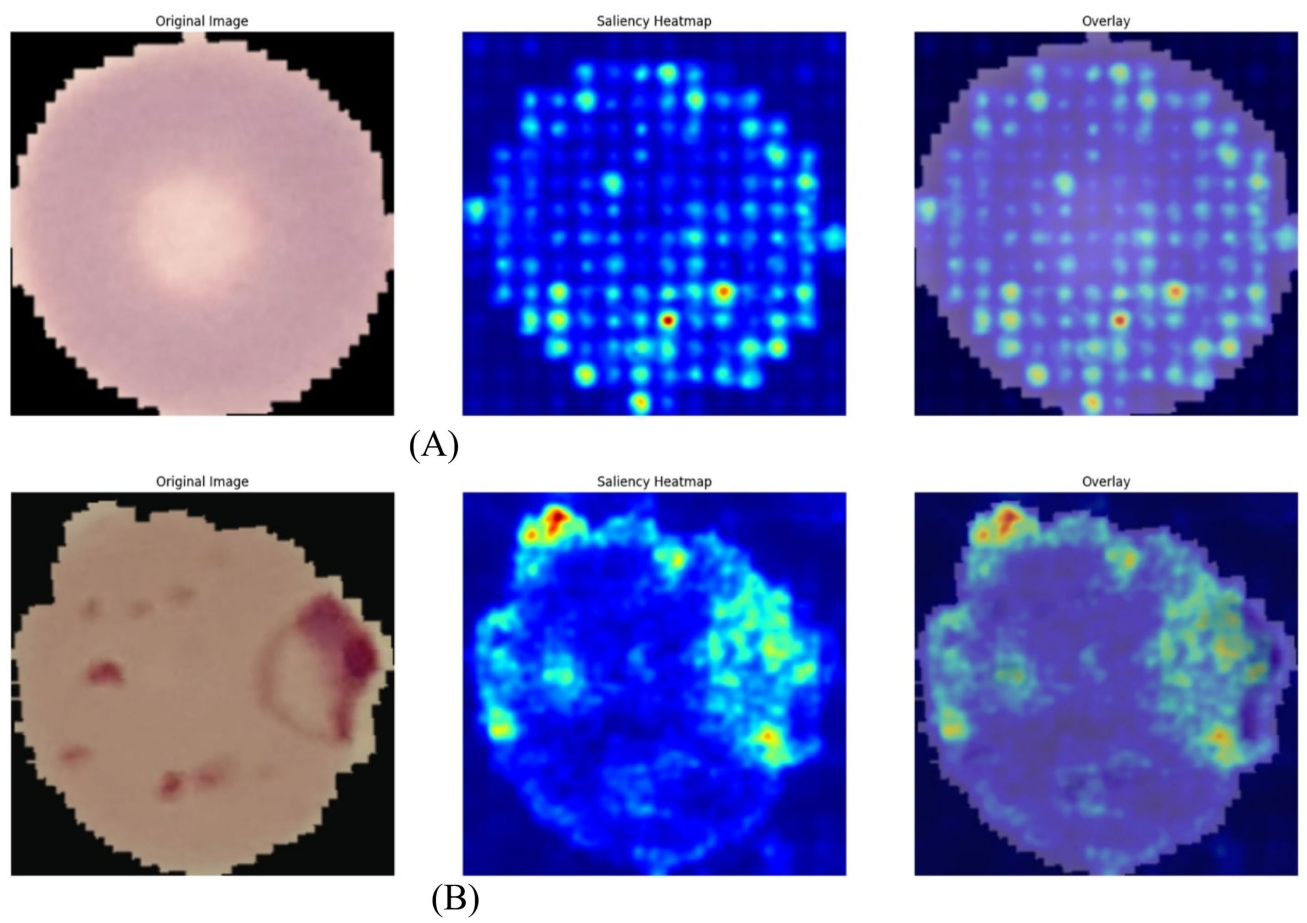
Comprehensive saliency map visualization—FedDistill-DeiT and FedAvg-Swin-T. **(A)** Saliency map of FedDistill-DeiT. **(B)** Saliency map of FedAvg-Swin-T.

Saliency maps across FedAvg models, including ViT-B/16, DeiT-Tiny, and Swin-T, consistently highlighted biologically relevant features such as trophozoites and schizonts, confirming accurate, data-driven predictions. FedDistill models reduced heterogeneity-induced noise, with FedDistill-Swin-T achieving an MCC of 0.956. FederatedDistill-DeiT heatmaps displayed high-intensity attention on parasite nuclei and abnormal tissue, enhancing diagnostic trust. These visualizations validated cross-client consistency and demonstrated effective decentralized attention allocation. DeiT’s data-efficient backbone generated precise and clinically reliable saliency maps, reinforcing the necessity of explainability and transparency in medical AI and aligning with contemporary research that emphasizes interpretable federated medical imaging for dependable clinical decision-making. Examples of these saliency maps are shown in Figure 6 for FedDistill-DeiT and for FedAvg-Swin-T.

In the federated learning setting, FederatedAvg-Swin-T saliency maps reveal attention distributed across diagnostically significant regions, shown from blue to red heat intensities. Swin Transformers surpass CNNs by integrating local and global context through hierarchical attention. The overlays highlight focus on abnormal tissues and cell boundaries, proving interpretability and clinical validity. Consistent visuals confirm successful aggregation, with FedAvg ensuring unified representation. These outcomes reinforce transformer-based explainable federated imaging as vital for reliable, privacy-preserving medical diagnostics across distributed clients.

### 5.2 Local model-agnostic explanations

To ensure a local interpretation, LIME provides an alternative approach whereby it constructs surrogate linear models on specific input instances (36). LIME examines every prediction of the federated transformer ensemble by training a locally faithful interpretable model 𝑔 to the target model image 𝑓 and yields perturbed input samples 𝑥′. The following is the definition of the optimization goal given in Equation 27:


$$\xi (g)=g\in G\;\text{argminL}(f,g,\pi_{x})+\Omega (g)$$ (27)

In order to guarantee model simplicity and interpretability, Ω(g) applies regularization restrictions, while $L(f,g,\pi_{x})$ quantifies the faithfulness of the interpretable model g to the original model f within the local neighborhood $\pi_{x}$ specified by a proximity measure.

**Figure 7:**
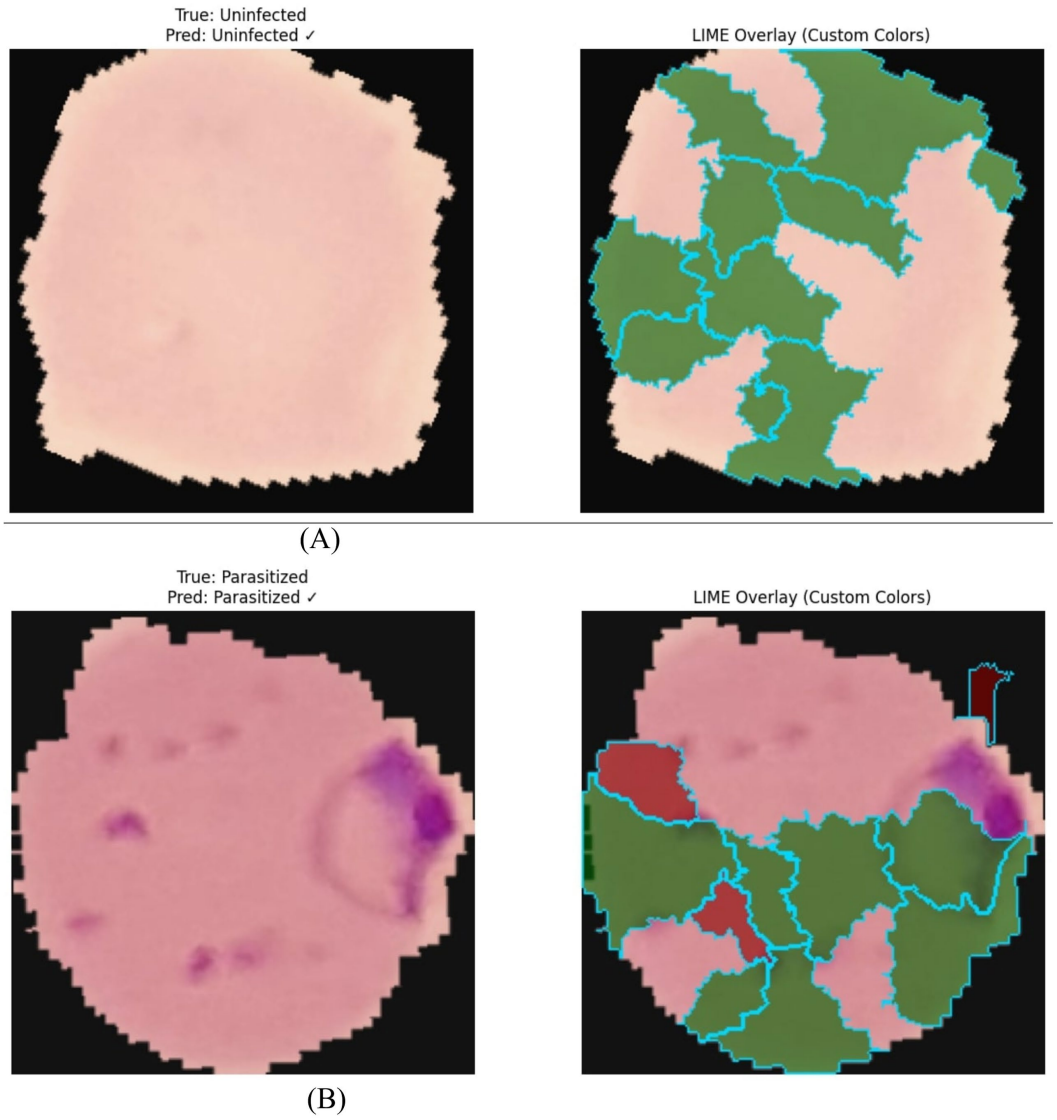
Comprehensive LIME visualization—FedDistill-DeiT and FedAvg-Swin-T. **(A)** LIME explanation of FedDistill-DeiT. **(B)** LIME explanation of FedAvg-Swin-T.

LIME succeeded in highlighting the key discriminative features for the FedDistill-Swin-T model: hemozoin deposits, membrane abnormalities, and cytoplasmic distortions, which are in perfect agreement with the strong F1-scores of 0.98 and MCC of 0.956, showing clinical relevance. DINOv2-based models maintained interpretability: FedAvg-DINOv2 with an MCC of 0.9444 and FedDistill-DINOv2 with an MCC of 0.9162. LIME reliably differentiated parasitized from healthy cells by pointing out pigment deposits, erythrocyte shape changes, and chromatin spots. In conjunction with saliency maps, it provides both pixel-level attribution and morphological insights to ensure transparent and accountable transformer-driven malaria diagnostics. The approach allows for robust and interpretable deployment in a federated learning setting across distributed clients and further confirms that knowledge distillation preserves critical diagnostic features while maintaining clinical reliability. LIME explanations illustrating this capability are presented in Figure 7 for FedDistill-DeiT and for FedAvg-Swin-T.

LIME explains the prediction of the FederatedAvg-Swin-T model on the parasitized red blood cell using green and red superpixels to highlight supporting and contradicting regions, respectively. Highlighting features like purple parasitic inclusions gives localized, gradient-free interpretability. Hierarchical attention and Federated Averaging together provide consistent clinically valid representations across clients. The confirmation of disease characteristics through alignment with highlighted LIME areas confirms diagnostic reliability. These findings emphasize the need for model-agnostic explainable AI in decentralized medical imaging supporting transparency, privacy, and robust deployment of federated learning.

Table 8 compares the proposed transformer-based federated learning architecture with classical malaria detection methods. The traditional models-VGG plus SVM hybrid by Vijayalakshmi (12), and ResNet-50 models by Rajaraman et al. (14); Reddy and Juliet (2019)-suffer from limited adaptability, insufficient regularization, and weak generalization. Though there was higher accuracy from MobileNet-V2 by Soylu (17) and DenseNet-201 by Çinar and Yildirim (15), these architectures have dataset imbalance, a higher computational cost, and lack of protection of privacy. Finally, the use of ViT-B/16, DeiT-Tiny, Swin-T, and DINOv2 combined with FedAvg and FedDistill in the proposed framework results in outstanding performance with the state-of-the-art results reaching as high as 97.79% accuracy and 0.9968 ROC-AUC, while preserving privacy. Along with saliency maps and LIME, it ensures interpretability, robustness, and balanced clinical performance, which enables reliable and large-scale malaria diagnosis in resource-limited settings.

**Table 8:** Comparison of proposed framework with existing methods.

Source	Model	Images	Accuracy (%)	ROC-AUC	Privacy-aware	Explainable
Vijayalakshmi (12)	VGG + SVM	2,550	93.10	–	No	No
Rajaraman et al. (14)	ResNet-50	27,558	95.90	–	No	No
Çinar and Yildirim (15)	DenseNet-201	6,730	97.83	–	No	No
Reddy et al. (2019)	ResNet-50	27,558	95.91	–	No	No
Soylu (17)	MobileNet-V2	27,558	96.53	–	No	No
Proposed (FedAVG-Swin-T)	**Transformer**	**27,558**	**97.75**	**0.9968**	✓	✓
Proposed (FedDistill-Diet-Tiny)	**Transformer**	**27,558**	**97.79**	**0.9966**	✓	✓

## 6 Limitations and future work

The present study demonstrates several constraints that warrant careful consideration and acknowledgment. The federated learning simulation was run on a single high-performance workstation instead of a group of geographically dispersed healthcare institutions. Such a centralized simulation does not adequately represent the network latencies, bandwidth limitations and overhead of communication that are inherent in a real-world multi-institutional deployment. Although they were comprehensive, the data were mostly obtained from controlled laboratory conditions using standardized staining protocols and imaging conditions. Clinical practice shows an even greater degree of variance in specimen preparation as well as imaging quality. Transformer models require a lot of computer power while training, so they could be limited in terms of direct implementation into a facility with severe resource requirements. Client heterogeneity was simulated through algorithmic partitioning of data rather than through actual inter hospital differences in patient demographics, parasite species distribution, and local patterns of disease prevalence. The alleged guarantees of privacy, though theoretically correct, have not been formally tested against model inversion attacks by sophisticated cryptanalysis. The investigation was limited to binary classification and did not include multi-class parasite species identification and multi-infection. Model interpretability was determined qualitatively through visual inspection of saliency maps, LIME explanations, as opposed to using quantitative faithfulness metrics across a spectrum of clinical cases. The above limitations underscore the need for thorough procedures of real-world validation before the widespread clinical implementation of such interventions.

Future work should focus on multi-institutional clinical trials in a variety of healthcare settings to ensure the robustness of federated models in a real-world operational setting. The combination of multimodal data that includes symptoms, biomarkers, and epidemiological metadata has the potential to significantly improve the accuracy of the diagnosis and to improve the contextual reasoning. Adaptive federated optimization algorithms that vary the weights of the aggregations according to some measure of client reliability and data quality, are a promising research avenue. The use of formal epsilon-delta guarantees on implementing differential privacy mechanisms would provide additional protection from emerging adversarial attacks. Development of light-weight model architectures that are optimized for the edge devices would allow for direct use on resource-limited microscopy devices in endemic regions. Interactive explainable AI interfaces that allow clinicians to query model decisions and get justifications on a real-time basis would enhance clinical trust and rates of adoption. Expansion to the multi-species detection of Plasmodium including *P. vivax*, *P. ovale* and mixed infections would increase clinical applicability. Federated learning framework will be evaluated and generalized with comparative research on other infectious diseases. This includes COVID-19 detection from chest radiographs and CT scans, Hepatitis B Virus through serological and histopathological data related to intrauterine transmission risks (37), and Human Papillomavirus through cervical cytology image screening for regional prevalence patterns (38). Research into the methods of continual learning contributes to accustoming to new strains of pathogens and reducing devastating forgetting. These improvements will help get federated learning closer to robust, equitable, and trustworthy global infectious disease diagnostics.

## 7 Conclusion

This work presents a privacy-preserving, federated learning framework with vision transformers for automatic malaria diagnosis, including DINOv2, Swin-T, DeiT-Tiny, and ViT-B/16. FedDistill methods outperformed the standard FedAvg, where FedDistill-DeiT achieved 97.79% accuracy, 0.9964 ROC-AUC, with strong MCC and Cohen Kappa, whereas FedAvg-Swin-T achieved 97.75% accuracy. The performance of DINOv2 implementations was competitive, with FedAvg-DINOv2 at 97.22%. Explainable AI techniques, including saliency maps and LIME, underlined morphologically significant features, hence confirming biological plausibility and clinical reliability. It balances the three key elements of accuracy, interpretability, and privacy. This makes it an extendable solution, particularly in resource-limited settings. Further research directions include the integration of multimodal data, real-world validation, adaptive deployment, and interactive XAI to establish and further improve the robustness of the results over a wide variety of medical imaging tasks.

## Data Availability

The raw data supporting the conclusions of this article will be made available by the authors, without undue reservation.
